# Impact of Maltitol and Sorbitol on Technological and Sensory Attributes of Biscuits

**DOI:** 10.3390/foods10112545

**Published:** 2021-10-22

**Authors:** Mathilde Roze, Doina Crucean, Guénaelle Diler, Cécile Rannou, Clément Catanéo, Camille Jonchère, Alain Le-Bail, Patricia Le-Bail

**Affiliations:** 1 ONIRIS, UMR GEPEA CNRS 6144, Rue de la Géraudière, CS 82225, 44322 Nantes, France; mathilde.roze@oniris-nantes.fr (M.R.); guenaelle.diler@oniris-nantes.fr (G.D.); cecile.rannou@oniris-nantes.fr (C.R.); clement.cataneo@oniris-nantes.fr (C.C.); alain.lebail@oniris-nantes.fr (A.L.-B.); 2 INRAe, UR BIA 1268, Rue de la Géraudière, CEDEX 3, 44316 Nantes, France; doina.crucean@inrae.fr (D.C.); camille.jonchere@inrae.fr (C.J.)

**Keywords:** biscuit, sugar replacement, water distribution, polyols, sensory properties

## Abstract

Overconsumption of sugars in diets is associated with many health problems, including dental diseases, diabetes and obesity. However, removing sugar from products such as biscuits is still a challenge for manufacturers and has been limited in Europe since the evolution of the EU regulation in January 2018, allowing only polyols and non-sweetening bulking agents as sugar substitutes. This study investigated the effects of fully replacing sugar with two polyols, maltitol and sorbitol, in short-dough biscuits. Morphological, textural and visual characteristics were studied as well as sensory properties. The reformulated biscuits were more compact in shape and structure. They were also less prone to checking, which was attributed to a more homogeneous water distribution at the end of baking, especially with sorbitol. Polyol biscuits were surprisingly colourful, especially sorbitol ones, although polyols are not normally involved in Maillard reactions. Sensory tests, however, showed a depreciation of the products compared to the control. Sorbitol biscuits were the least preferred but maltitol ones were quite well accepted compared to the control. Thus, maltitol is an excellent potential substitute for this type of product.

## 1. Introduction

Alongside flour and fats, sugar is a key ingredient with important functions in biscuits [1]. Beyond sweetness, it is responsible for the characteristic golden-brown colour of the biscuit and aromatic notes through caramelization and Maillard reactions [2]. Sugar also participates in the structural and textural properties [3]. Unfortunately, the direct and indirect impacts on the health of a diet rich in sugars represent an increasingly important burden for our societies: diabetes, obesity, dental and cardiovascular diseases [4,5]. Restricting our sugar consumption has become an imperative: health authorities have formulated numerous recommendations on the consumption of sugars and sugar-vectoring products such as biscuits, sweetened drinks or confectionery [4,6], while public authorities have initiated policies to encourage industries to improve the nutritional profile of these products. Sugar removal in biscuits is often hindered by technological constraints and also by a sharp sensory depreciation. Since January 2018, the use of intense sweeteners has been prohibited in biscuits [7]. Only sugar alcohols and non-sweetening bulking agents are authorized. As biscuits are gourmet products, consumers expect a minimum of sweetness, making the use of non-sweetening bulking agents more complicated. Among polyols, not all of them fulfill the required functions beyond the “bulk function”. For example, lactitol and isomalt are significantly less sweet [8,9]. Erythritol and xylitol have an unexpected and rather undesirable cooling effect in baked goods due to a very negative heat of solution [9], while mannitol is a poor candidate for substituting sugar in cereal products [10].

This work aims to develop biscuits without added sugars in which sucrose has been fully replaced by maltitol and sorbitol. These two polyols have suitable properties to substitute sugar in baked goods but have different characteristics. Maltitol presents characteristics close to sucrose, with similar molecular weight (disaccharide), melting point and hygroscopicity, while sorbitol is less hygroscopic, smaller (monosaccharide) and melts at lower temperatures. Although no-added-sugar biscuits may be of great interest to populations suffering from diabetes or wanting to monitor their caloric intake, only a few attempts have been made to completely substitute sugars either with sorbitol or maltitol [11,12,13]. In particular, to our knowledge, no study has investigated the impact of sugar replacement by polyols on checking and on water distribution in biscuits. Checking propensity is a main quality concern for biscuit manufacturers. This condition is usually considered through process modifications in the literature [14,15], while little work has been done on formulation. As previous work has established the link between checking and water distribution [16,17], we chose to fully replace sugar with two polyols whose physicochemical characteristics presuppose different interactions with water. The sweetening powers of maltitol and sorbitol are also different. Maltitol is almost as sweet as sucrose (sweetening power 0.9), while sorbitol is slightly less sweet (sweetening power 0.5). Replacing sugars in biscuits is only of interest if the resulting products remain appreciable by most consumers. In this context, sensory tests were conducted following the instrumental analysis. 

Therefore, this study chooses to study both the technological and sensory impacts of the substitution of sugars in biscuits by two polyols (maltitol and sorbitol) and provide a specific light on the effects on checking and water distribution in the product. The objective of this work is in particular to provide avenues for improvement to manufacturers wishing to improve the nutritional profile of their products or to develop biscuits intended for people with nutritional constraints.

## 2. Materials and Methods

### 2.1. Materials

#### 2.1.1. Ingredients

Ingredients used in dough preparation were Type 65 white wheat flour (Moulins Evelia—Terrena, Orée-d'Anjou, France) (14.7% moisture, 9.5% protein, 0.61% ash), butter (Président, Lactalis, Laval, France), salt, tap water, sodium bicarbonate (Brenntag, Saint-Herblain, France) and disodium pyrophosphate (Chemische Fabrik Budenheim KG, Budenheim, Germany). Either granulated sucrose (Béghin Say, Tereos, France), maltitol (Louis François, Croissy-Beaubourg, France) or sorbitol (Louis François, France) were used as sweeteners (full replacement on weight basis). Formulations are presented in Table 1.

#### 2.1.2. Dough and Biscuit Preparation

All ingredients were stored at 23 °C the day before production to ensure similar temperatures for each production except for butter put to thaw 14 h before use from −18 °C to +15 °C. Butter, leavening powders and the sweetening agent were creamed with hot water (50 °C) in a kneader (BV422, VMI, France) at 62 rpm for 1 min and 125 rpm for 2 min. Flour was added and mixed with the rest at 62 rpm for 1 min, then at 125 rpm for 2 min. The average temperature of the obtained doughs was 21.7 ± 1.6 °C. Dough was left to rest for 30 min at 30 °C before forming biscuits with a rotary moulder (R2, Padovani, Rovigo, Italy). Biscuits were baked in a ventilated oven (Premio, Bongard, Holtzheim, France) for 9 min at 210 °C. Six trays of 40 biscuits were placed in the oven. Dough pieces were round-shaped, 60 mm in diameter and 4 mm in thickness. Each batch resulted in 240 dough pieces with an average individual mass of 12.0 ± 0.3 g. Following baking, biscuits were transferred onto plastic trays and placed in polystyrene boxes to cool for 1 h. The biscuits were kept in hermetic boxes during storage.

### 2.2. Biscuit Quality

#### 2.2.1. Colour

The colour of 15 biscuits was measured 24 h after baking with a colorimeter (CR-400, Konica Minolta, Orvault, France) at the centres and edges of the upper and lower surfaces of the biscuits. Biscuit colour was given according to CIE system variables L* (lightness), +a* (redness) and +b* (yellowness). The colorimeter was calibrated with the supplier’s white tile and the parameters were: aperture = 8 mm, standard observer 2°, and illuminant D65. Visual colour difference Δ*E* between control and reformulated biscuits was calculated between the average L*a*b* values from each location according to the formula:

Δ*E* = [(L_2_* − L_1_*)^2^ + (a_2_* − a_1_*)^2^ + (b_2_* − b_1_*)^2^]^1/2^, (1)

As a rule, if Δ*E* < 1, no colour difference is apparent to the human eye. Between 1 and 3, minor colour differences may be perceived depending on the hue and eye sensitivity, while colour differences are evident when Δ*E* > 3 [18].

#### 2.2.2. Biscuit Morphology

The thickness and diameter of 15 dough pieces were measured with a digital calliper (Mitutoyo, Japan) 10 min after being moulded. Measurements were taken with two orientations at a 90° angle difference to mitigate any artefact from orientation effect caused by the rotary moulder. The parameters were measured again on corresponding biscuits 24 h after baking. Thickness gain is defined as the difference between a biscuit and corresponding dough piece thicknesses and is expressed as a mean value ± standard deviation.

The shrinkage index (S) was computed from dough piece diameter (D_raw_) and corresponding baked biscuit (D_baked_):

S = 100 × (D_raw_ − D_baked_)/D_raw_. (2)

#### 2.2.3. Checking

For each production, 30 biscuits were collected after cooling for 1 h and stored on impression plates to not be shaken during storage. Cracks were visually monitored between 1 h and 8 h after cooling, then once a day for the first seven days and finally 28 days after production. Checking monitoring was made in triplicate (three batches per recipe). Results are expressed as mean checking ratios (proportion of cracked biscuits) ± standard deviation.

#### 2.2.4. Hardness

Hardness was measured with a three-point bending test using a texturometer (TA1, Lloyd Instruments Ametek S.A.S., Elancourt, France) provided with Nexygen Plus software. Hardness was measured 1 h and 2 h after baking on biscuits, before crack formation. Test parameters were 1 kN force sensor, 40 mm spacing between supports, 0.5 N preload, 30 mm/min preload speed, 50 mm/min speed. Hardness is defined as the maximum breaking force (N).

#### 2.2.5. Biscuit Porosity

Biscuit porosity (Pt) was computed according to the following formula:

Pt = 1 − ρ_bulk/_ρ_solid_, (3)

with bulk density (ρ_bulk_) defined as the mass/volume ratio of a solid (pores included) and solid density (ρ_solid_) defined as the mass/volume ratio of the pure solid. The bulk density was measured using a modified Archimedean method with rapeseeds of known density [19]. Measurements were repeated five times. Solid density was measured 10 times for each biscuit using an automatic helium pycnometer (Accupyc 1330, Micromeritics, Norcross, GA, USA). Porosity values are expressed as percentages.

#### 2.2.6. Water Distribution

Dough water content was determined right after kneading. Nine samples of 4–6 g from three different batches were collected in foil pans and dried in a cabinet at 105 °C for 24 h. During the production, the total water content (free and bound water) was measured in biscuits using a Karl Fisher titrator (V30S, Mettler Toledo, Columbus, OH, USA). Each sample was placed in a sealed vial. Vials, seals and seal protections were weighed before production and after sampling to deduce the exact sample weight after production time. This method avoided the long process of weighing each sample during production. Thus, sampling was nearly instantaneous and samples were prevented from drying out. The thinness and friability of the biscuit did not allow it to be repeatably separated into horizontal layers (inner layer and upper and lower outer layers). Plus, the water content gradient along the thickness of the biscuits was expected to be much lower than along the radius direction; therefore, the choice was made to sample along the radial axis. Six biscuits from two batches per recipe were punctured in the centre and periphery. Sampling times were: T0 + 10 min, 20 min, 30 min, 40 min, 50 min, 1 h, 1 h 30 min, 2 h, 3 h, 4 h, 5 h, 6 h, 7 h, 8 h, 24 h, 48 h and 7 days with T0 being time when the biscuits were removed from the oven.

#### 2.2.7. Temperature History of Biscuits

Oven temperature and the core temperature of three biscuits were recorded during each batch from baking to storage with a data logger (OCTTEMP2000, Omega, Manchester, BD, UK) and four type-K thermocouples. This monitoring makes it possible to check whether the three doughs behave in the same way and therefore follow the same temperature kinetics during baking and cooling.

#### 2.2.8. Experimental Design and Statistical Analysis

Each formulation was produced in triplicate. Colour, dimensions, and porosity were measured on 15 biscuits taken from the three batches while hardness was measured on nine biscuits. Checking rates were measured on 90 biscuits sampled from the three batches. Karl Fisher measurements were made on six biscuits from two different batches. Only biscuits at the centre of each tray were used in analyses to avoid baking heterogeneity at the sides of the oven. Results are presented as mean values ± standard deviation. Statistical significances are computed with a Duncan test (95% level significance) after performing a one-way ANOVA using R software (version 3.6.2) [20] and *agricolae* R package (version 1.3.3) except for sensory analysis where a two-way ANOVA was performed (panelist, formulation).

### 2.3. Sensory Evaluation

The sensory tests were conducted in accordance with the Declaration of Helsinki [21]. All applicable institutional and governmental regulations concerning the ethical use of human volunteers were respected during this study. Sensory tests were approved by the ethics evaluation committee of the French Institute of medical research and Health (IRB00003888, IORG0003254, FWA00005831). A panel of 62 untrained sensory assessors (students and employees of Oniris) evaluated the biscuits. Participants were informed of the study’s purpose and signed a consent form.

Tests took place in a sensory analysis room equipped with individual booths, standardized light and temperature. One biscuit from each formulation was presented in a random order in a cardboard liner. Biscuits were coded with random three-digit numbers. All recipes were produced 3 to 4 days before the tests. Panelists had mineral water (Evian) at their disposal while tasting the biscuits. Overall liking was assessed with a nine-point hedonic scale (dislike extremely = 1, neither like nor dislike = 5, like extremely = 9). Next, a JAR test (Just About Right) with a 5-point scale (1 = not nearly enough, 3 = just about right, 5 = much too) was used to evaluate colour, hardness, general aroma intensity, sweetness and compactness. Overall liking scores were aggregated into three classes: dislike (ratings ≤ 4), neither like nor dislike (ratings = 5) and like (ratings ≥ 6). Mean liking scores, standard deviation and significances (α = 0.05) were calculated for each formulation. Similarly, JAR ratings were aggregated into the following groups: not enough (ratings 1 and 2), just about right (ratings 3) and too much (ratings 4 and 5). Penalty analysis was applied to the results (XLSTAT, Addinsoft (2021), Paris, France).

## 3. Results and Discussion

### 3.1. Dough Characteristics

#### 3.1.1. Physical Measurements before Baking

The dough pieces were similar in shape for the three formulations (Table 2). No significant difference was observed concerning the thickness. However, maltitol dough pieces had a smaller diameter. Indeed, the more crumbly dough appeared less able to pass through the rotary moulder causing minor defects in the diameter of the dough pieces without affecting the product’s properties. Zoulias et al. [11] characterized in-depth the texture parameters of doughs formulated with polyols and showed no difference between sucrose and sorbitol regarding adhesiveness, consistency and cohesiveness while maltitol dough exhibited lower adhesiveness and cohesiveness and a slightly higher consistency but with no apparent consequence on handling according to the authors.

#### 3.1.2. Water Content

All doughs had water contents of the same order of magnitude, as shown in Table 3. Sorbitol dough, however, was slightly more hydrated than the control and maltitol ones. All sweeteners used in the study did not initially have the same water content. Indeed, the water content in sucrose and maltitol were respectively 0.05 ± 0.01% and 0.041 ± 0.003% while the water content in sorbitol was 0.22 ± 0.02%. This could partially explain the slightly higher water content of the dough with this substitute.

### 3.2. Biscuit Physical and Textural Characteristics

#### 3.2.1. Biscuit Morphology

- Thickness

During baking, air bubbles incorporated into the dough during creaming, expand and eventually coalesce. The expansion during baking is caused by gas expansion, water vaporisation and CO_2_ production by the leavening agents. At the end of baking, a porous and dry structure is obtained. After baking, the thickness of control biscuits was significantly higher (+9%) than those of sorbitol and maltitol biscuits (Table 2). However, no difference was observed between the different polyol formulations. These observations match with the results from Zoulias et al. [11] whose maltitol and sorbitol biscuits had a thickness of 6.5 mm while sucrose biscuits were 7.5 mm thick (+13%). Polyols could interact differently during baking and impact the setting time of the biscuit during baking or follow a different core temperature kinetics, resulting in a lower expansion compared to the control biscuit. Monitoring the temperature of the biscuits did not confirm this hypothesis. During the manufacturing process, biscuits were baked for 9 min in the oven at 210 °C. When entering the oven, all biscuits underwent a rapid rise in temperature reaching 105–115 °C within 2 min, followed by a more progressive rise until reaching its maximum at 155–160 °C. Once removed from the oven, the centre temperature of the biscuits dropped to 90 °C in 5 min. From 50 °C (8 to 15 min after baking), the temperature drop was slower, and biscuits reached room temperature within 1 h. All recipes followed the same temperature kinetics during baking suggesting heat transfer rates where similar in each formulation. Still, interactions between polyols and other constituents would be liable to affect the protein denaturation and starch gelatinization temperatures with consequences on the structure and dimensions of biscuits. Beyond thickness, control biscuit morphology was also different with a slightly domed centre. Despite this curvature, the centre thickness of the control biscuit was not affected when compared to the edges.

- Shrinkage

Biscuits may exhibit a small shrinkage during baking. This phenomenon indicates a probable partial development of the gluten network during production, although this is not desired with biscuits. This phenomenon can occur during mixing or rotary moulding and is linked to water availability. In this study, mixing is purposely done over a short time in two stages, creaming then kneading. The addition of the flour during kneading allows a shorter mixing time, resulting in a cohesive and homogeneous dough being obtained and a low level of gluten network development [22,23,24]. However, forming dough pieces with a rotary moulder subjects dough to significant mechanical stresses. When the gluten network starts to develop, the dough gains some elasticity and resulting dough pieces shrink when baked. A slight shrinkage is observed with polyol doughs, respectively 1% and 1.2%, while no significant shrinkage was observed for the control one. Sucrose could further restrict the gluten network compared to polyols, or could counteract the effects of gluten network development by the spreading it usually generates during baking [25].

#### 3.2.2. Colour

Biscuit crust colour was measured in four locations to reflect potential colour heterogeneity between the top and the underside and between the centre and the edges. L*a*b* values and colorimetric distances between control and polyol biscuits are given in Table 4.

The control biscuit showed heterogeneity in colour between the centre and the periphery on its two surfaces. The biscuit’s centre was lighter in both cases. During baking, the temperature rose faster in the periphery compared to the centre. The biscuit dehydrated faster and baked more in this area, resulting in a darker colour. The colour difference between the centre and the edge was greater on the underside, where the centre was extremely pale. This lighter colour was attributed to the presence of an air gap, which appeared during baking, between the dough piece and the baking tray. Maillard and caramelization reactions generating the colour did not occur in this area. This phenomenon was not observed on sorbitol biscuits and was attenuated on maltitol ones. Polyol biscuits also showed heterogeneity between the centre and the edge on both sides. On the upper side, both substituted biscuits exhibited small differences with the control in the centre (Δ*E* < 3). On the periphery, the difference remained small for maltitol. However, sorbitol was much darker. On the underside, differences were more pronounced, particularly in the centre, since the reformulated biscuits did not present any air gap. Again, sorbitol biscuits were darker than the control ones. Around the edges, the colour difference compared to the control was visible for both polyol biscuits. Maltitol biscuits were particularly lighter.

The biscuit’s colour results from Maillard and caramelization reactions taking place during baking. As the temperature rises, sucrose may hydrolyze into fructose and glucose, which take part in these reactions. Maltitol and sorbitol do not have the reducing function necessary to participate in these reactions. It, therefore, appears unexpected that sorbitol and maltitol biscuits are, after all, exceptionally colourful. Sorbitol biscuits especially were darker and redder than maltitol ones, suggesting other reactions occur in the sorbitol biscuits during baking or occur more intensely. The biscuits’ temperature monitoring showed the sorbitol biscuits had not undergone a different temperature kinetics than the other ones. Therefore, the difference in colour brightness does not come from a faster or higher temperature rise. Ronda et al. [26] observed a slightly lighter crust for sorbitol cake and much lighter for maltitol cake compared to a sucrose one. Conversely, Psimouli and Oreopoulou [27] did not observe lightened crusts in cakes reformulated with maltitol or sorbitol. Although Ronda et al. [26] did not discuss it explicitly, their results also showed a colour difference between maltitol and sorbitol, maltitol producing lighter cakes.

#### 3.2.3. Checking

**Figure 1:**
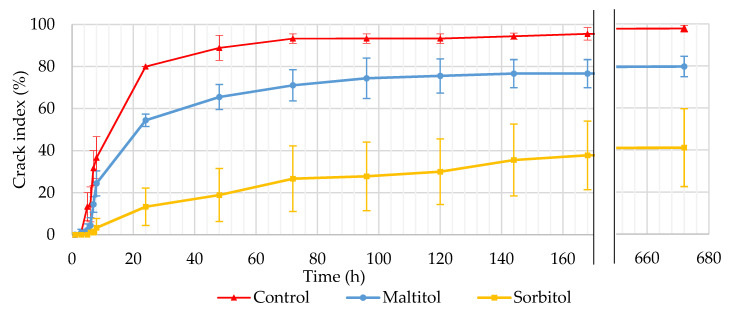
Evolution of crack index expressed in % and monitored on biscuits from the end of baking (T0) to 28 days (n = 90).

Biscuit checking is a condition observed through the appearance of a small hairline crack at the biscuit’s surface during cooling or storage. This crack usually lengthens from the centre towards opposite edges and thickens as it goes deeper to the biscuit’s core. Cracks dramatically increase biscuit fragility and may lead to breakage. As breakage occurs at the end of line production or is discovered by consumers when opening packaging, it can be associated with the dead loss for the manufacturer and consumer discontent. The proportion of cracked biscuits was monitored within 8 h after baking, then for 7 days and lastly on day 28 (Figure 1). The recipe studied in this work was specifically selected as it produces high checking rates when unique baking is applied without a second thermal treatment in the manufacturing process. In our study, cracks began to appear on the underside in the centre of biscuits and extended towards two opposite edges. Seldomly, they extended in three to four directions. Over time, the line lengthened, deepened and widened. In some cases, it reached the upper surface and sometimes breakage occurred.

Cracks appeared quickly on the control biscuit, as early as 5 h after baking. Checking reached a significant rate (36.7%) at T0 + 8 h. After 24 h, 80% of the control biscuits showed cracks and from 72 h, the checking rate began to level off at around 93–96%. At 28 days, 97.8% of the biscuits were cracked and some were spontaneously broken. These results are consistent with checking rates obtained by Bedas et al. [17] on biscuits with a comparable recipe and process but slightly different thicknesses. Maltitol biscuits appeared to be less susceptible to cracking although checking kinetics are similar to that of control ones. At T0 + 24 h, 54.4% of the biscuits were cracked, and the crack rate stabilized at 74–76% between 4 and 7 days. The crack rate reached 79.7% at 28 days. Sorbitol biscuits were less prone to cracking. At T0 + 24 h, the crack rate was 13.3%. The crack rate gradually increased throughout the first 7 days to reach 37.8%. There was no real plateau, unlike the other two formulations. However, at 28 days, the crack rate had increased only slightly (41.1%). The variability of the sorbitol recipe was higher than that of the other formulations. The polyol recipes were less subject to checking than the control, but in different proportions. This difference in behaviour was noticeable over the first hours, even before the biscuit started to equilibrate with its environment. To our knowledge, this particular effect of polyols has not been shown before on cereal products.

Previous work has established the link between checking and the existence of a water gradient within the biscuit [14,16,17]. By comparison of cookies baked in a forced convection oven and others baked in an oven heated by near-infrared radiation, Wade [14] showed a higher homogeneity in the biscuits’ residual moisture and no signs of checking when using near-infrared radiations. Ahmad et al. [15] have done similar work using microwave post-baking. Checking rates of microwave post-baked biscuits were significantly lower (5%) compared to convection-oven baked biscuits (61%) while both had a similar final water content. To identify the origin of these differences, the distribution of water was therefore studied over time to confirm the existence of a center-edge humidity gradient within the biscuits in our study. If so, the specific properties of the polyols with respect to water could be the cause of this reduced tendency to checking observed on reformulated biscuits.

#### 3.2.4. Hardness

Hardness measurements were done on nine cooled biscuits using the three-point bending method. Results in Table 5 show that the control biscuits were softer than the substituted recipes one hour after baking. However, the difference was not statistically significant afterward. No difference in hardness is observed between maltitol and sorbitol biscuits. Standard deviations are high but resulting variance coefficients are in the range of those obtained in the literature on similar products [28]. Sahin et al. [29] observed an increase in hardness when sugar is partially substituted with maltitol or sorbitol in other cereal baked products. On the contrary, Zoulias et al. [11] did not observe any difference in hardness between sucrose and maltitol biscuits and observed a softer texture with sorbitol. Laguna et al. [13] did not find any difference in hardness between sugar and maltitol with the same method. Ronda et al. [26] and Psimouli and Oreopoulou [27] observed no difference in cake firmness between sucrose and maltitol or sorbitol recipes.

#### 3.2.5. Porosity

Biscuits had porosities ranging between 50% and 55% (Table 5). These values are slightly higher than those obtained by Pareyt et al. [30] on sugar-snap cookies and slightly lower than the results of Mieszkowska and Marzec [31] on short-dough cookies, although recipes contain, respectively, more sugar and more fats and sugar. Control biscuits were more porous than the polyol biscuits. This could indicate that less air was incorporated into the dough during creaming. Consequently, fewer and/or smaller air bubbles developed during baking, leading to a more compact biscuit. These results confirm the tendencies observed in hardness analysis. The control biscuit is more porous and consequently less hard than substituted biscuits.

### 3.3. Checking Origins: Water Distribution

**Figure 2:**
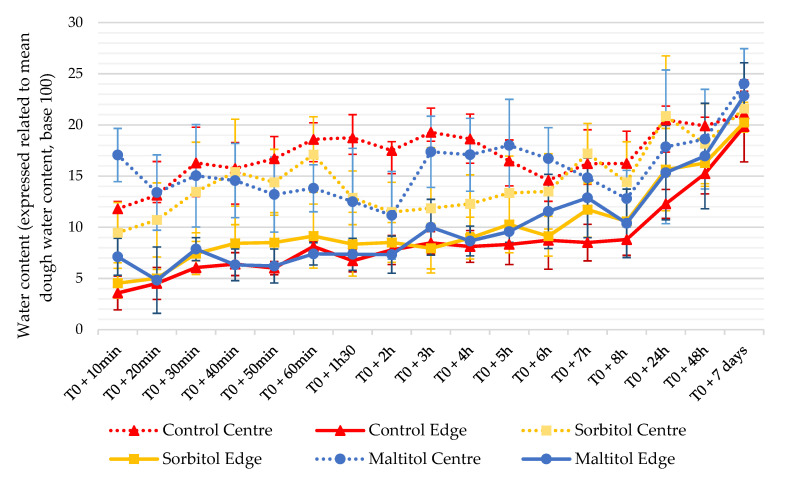
Evolution of water content in the centre and periphery of biscuits from end of baking to 7 days (n = 6). Water contents are expressed related to the mean dough water content (base 100). T0 corresponds to the end of baking.

Water content was determined in the centre and periphery of biscuits from cooling to storage (Figure 2). During baking, water evaporates in the biscuit. This evaporation is not isotropic, with a higher evaporation at the edge than in the centre. During baking, the temperature rise occurs from the periphery to the core of the biscuit; hence, the periphery heats up faster and undergoes more intense drying since the temperature is higher for a longer time in this zone. Water evaporates and conversely escapes from the core to the periphery. At the end of baking, biscuits had a very low water content at the edges and higher in the centre. For all formulations, moisture contents are different between the centre and the edge of biscuits. In the hours following baking, water content is lower at the edge regardless of the recipe. From 24 h, the water content increases in the centre and edges without stabilizing until the end of the measurements.

**Figure 3:**
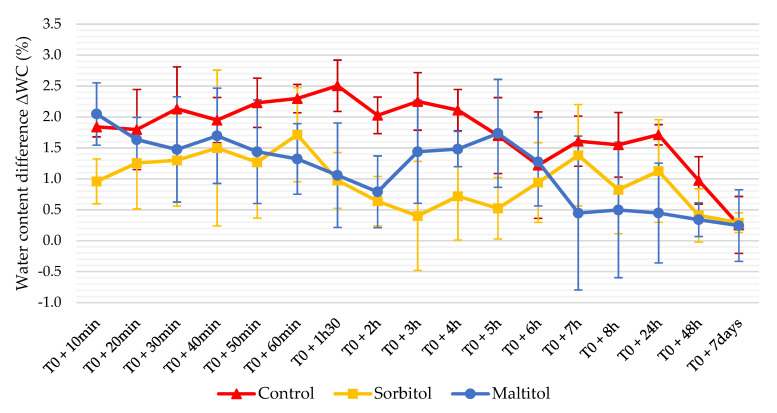
Evolution of water content difference ΔWC between centre and periphery of biscuits (n = 6). T0 corresponds to the end of baking.

Figure 3 shows the evolution of water content difference (ΔWC) between the centre and the edge. Control biscuits have the highest ΔWC over the first few hours with values between 1.8% and 2.5% (d.b.). After 5 h, their ΔWC decreases and stabilizes around 1.5% before dropping below the threshold of 1% at 48 h. Maltitol biscuits exhibited a lower ΔWC between 0.7% and 2% up to 6 h after baking when it falls below 1.5%. Sorbitol had the lowest ΔWC with values systematically at the 1.5% threshold or at the limit. A gradual increase of water content in the biscuit periphery was observed over 8 h following baking for all recipes. This kinetics is the most obvious for the sorbitol biscuits. Next, a general water uptake of the biscuit was observed. This intake of water from the environment happens in the centre and, to a greater extent, at the edges of biscuits. Bedas et al. [17] obtained comparable results on a similar control recipe with biscuits of different geometry or thickness. At 7 days, the water gradient had almost vanished (ΔWC = 0.25) and was identical between formulations. However, one might consider that biscuits are in equilibrium from 24 to 48 h after baking with a gradient close to 1%. Conversely, the biscuits did not seem in balance with their environment since their overall water content continued to increase until the end of the experiment.

Water gradient is important to consider when talking about checking. This gradient is one of the main phenomena at the origin of cracks [14,16,28]. Previous work has already shown that a high ΔWC, especially in low-fat short-dough biscuits, is associated with checking [17,32]. This ΔWC leads to water transfers within the biscuit, which tends to equilibrate from the centre to the periphery and from the surrounding environment to the surface [33]. This results in a hygroscopic expansion of the peripheral zones [34] and a contraction of the central zone, losing moisture. These dimensional changes cause tensions within the biscuit that weaken it and may eventually lead to the appearance of cracks. The heterogeneity in moisture was lower in sorbitol biscuits, suggesting the water balance was reached faster. Specifically, the ΔWC in the sorbitol recipe remained around 1%, which is the threshold value for the appearance of cracks in the work of Bernussi et al. [32]. Water movements are, therefore, of lesser magnitude compared to the control biscuit, whose water gradient was maintained at higher values for a longer time. Consequently, generated strains were lower and tolerated by a higher proportion of biscuits. Conversely, tensile strains within sucrose biscuits were greater and consistently exceeded the rupture stress. The strain tolerance of the biscuit depends on whether it is in its glassy or rubbery state. In the glassy state, the ability of the biscuit to undergo deformation under stress is reduced and it is more prone to breakage. Thus, these results are consistent with crack rates measured previously, sorbitol being the least susceptible to checking while the control presents a much higher propensity.

### 3.4. Sensory Acceptance

#### 3.4.1. Overall Product Appreciation

**Figure 4:**
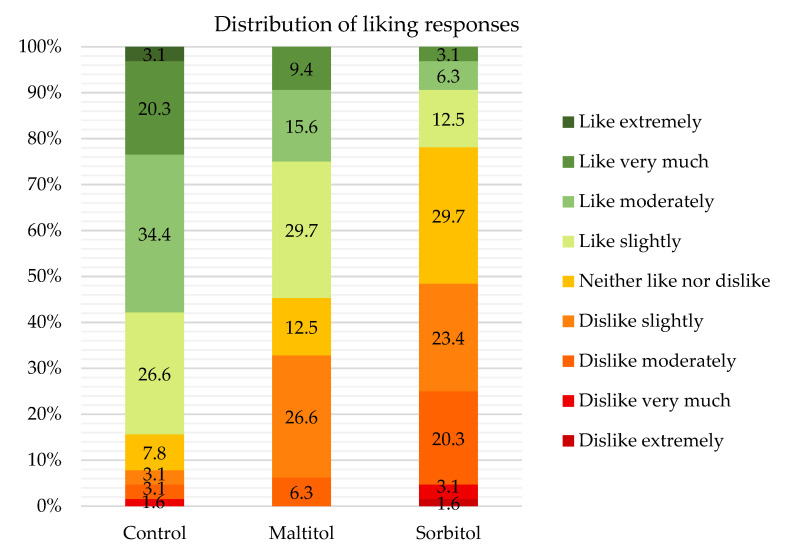
Percentage of respondents for the nine hedonic categories (1 = dislike extremely, 5 = neither like nor dislike, 9 = like extremely), (%, n = 62).

The control showed a mean hedonic rating of 6.6/9 (Table 6). As illustrated by Figure 4, most panelists (>80%) liked it and less than 8% disliked it. Maltitol showed a fair liking score of 5.5/9. Although it was less appreciated than the control, slightly more than half of the panelists liked it while about a third disliked it. The control and maltitol were, therefore, rather appreciated. Sorbitol was the least accepted product with a score of 4.6/9, which is just below average. So, this formulation can hardly be qualified as appreciated. Sugar substitution by the two polyols significantly decreased biscuit liking. This effect was more pronounced for sorbitol.

#### 3.4.2. Evaluation of Attributes

**Figure 5:**
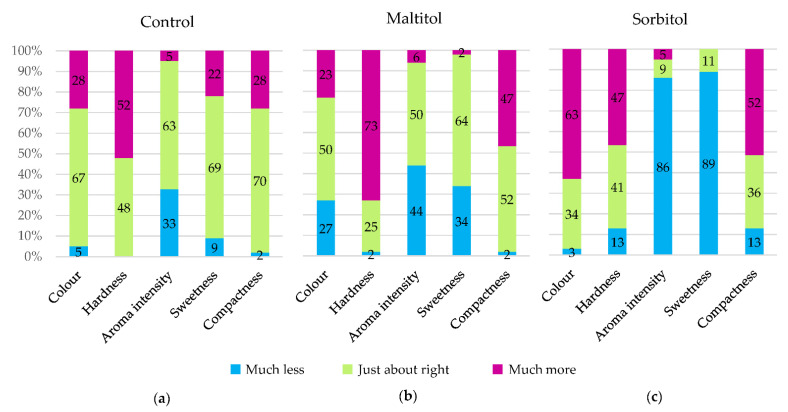
Just-about-right (JAR) scale percentages of responses aggregated in three levels (“Not enough”, “Just about right”, “Much too”) of the control (**a**), maltitol (**b**) and sorbitol (**c**) biscuits (n = 62).

In Figure 5, the JAR test results for the control show that each attribute is JAR for a majority of judges (>60%) except for hardness which was too much for half of the panelists. For maltitol, colour, aroma intensity, sweetness and compactness were JAR for half of the panelists. However, it was perceived as too hard (70% of judges) and too compact (47%). A lack of aroma intensity and sweetness was pointed out by more than a third of the judges. For sorbitol, none of the attributes was JAR for a majority of panelists. The biscuit was too dark and too hard for respectively 63% and 47% of panelists while aroma intensity and sweetness were not enough for over 85% of panelists. The biscuit was too compact for half of the panelists.

Results showed that the control was closest to the ideal biscuit, while sorbitol did not seem to perform as well. Maltitol seems to be a good challenger from a sensory standpoint, but produces biscuits perceived as harder. None of the attributes assessed in the JAR had a significant effect on the product liking mean.

The dark colour of sorbitol biscuits measured with the colorimeter seems indeed to be perceived by the judges, since 63% of them find it too dark compared to less than 30% for the other two recipes. Colour thus seems to be particularly important to increase consumers’ acceptance of the biscuit and needs to be carefully controlled.

Although no difference in hardness was found by instrumental measurements between maltitol and the other two recipes, more judges found it too hard. About half of the panelists found the polyol biscuits too compact, while the control was mostly JAR. This result is in agreement with the porosity values measured on products: the control is more aerated. Research to reduce the hardness of the polyol biscuits has to be conducted to reach consumers’ expectations.

The theoretical sweetening powers of maltitol and sorbitol are 0.9 and 0.5 compared to sucrose (sweetening power = 1) [8]. Control and maltitol biscuits were perceived as JAR for most panelists. The control is fairly balanced between not enough and too sweet, while maltitol is considered not enough by a higher proportion of panelists due to its slightly lower sweetening power. A large majority of panelists, on the other hand, considered sorbitol insufficiently sweet. This result was logically expected as the loss of sweetness was not compensated by other means in the product. In the work of Zoulias et al. [11], a trained panel also rated the sweetness in maltitol and sorbitol biscuits, respectively, as slightly lower and much lower compared to the control. Sorbitol biscuits were also perceived to be less aromatic, while no difference was observed between maltitol and sucrose.

## 4. Conclusions

The effects of sugar replacement on texture, appearance and structure demonstrate the importance of the functions it performs within the biscuit. However, substituting sugars in biscuits and other food products is still very relevant in the context of reducing chronic diseases. This work aimed to provide insights to manufacturers who engage in the process of improving the nutritional profile of their products. Replacement with polyols led to minor differences in dimensions but produced more compact and visually different biscuits. The biscuits were comparatively lighter for maltitol and darker for sorbitol.

Interestingly, biscuit fragility was also modified. Particularly, sorbitol was associated with biscuits which were less susceptible to crack thanks to a more even water distribution within the product. Few differences were observed, however, with maltitol on these aspects. Further studies on the interactions between these polyols, water and starch could be of interest with regard to the characterization of the biscuit’s propensity to checking. Sugar substitution in biscuits resulted in a lower acceptance from consumers. Sorbitol biscuits were not particularly appreciated, perceived as not sweet enough nor aromatic enough.

Conversely, the maltitol biscuits were rather well appreciated, almost as much as the control. Yet, the sweetness and aroma intensities were a little lacking and the biscuits were perceived as too hard. In this context, our work allows us to conclude that maltitol is the most likely to be accepted by consumers of the two polyols tested. In particular, there is little work for it to compete with sucrose in a food product like biscuit. The increasing sweetness could improve the sensory performance of reformulated products. However, high-intensity sweeteners are not authorized in biscuits [7]. Other strategies involving multimodal interactions or heterogeneous redistribution of the sweetening agent should, therefore, be considered. Indeed, reformulating a biscuit to improve it nutritionally remains a challenge: all modalities (aroma, taste, texture, appearance) must be taken into account for this new product to be accepted by consumers. In particular, the sensory profile remains a factor that cannot be sacrificed in gourmet products such as biscuits.

## Figures and Tables

**Table 1:** Dough formulations (%, wet basis).

Ingredients (%, Wet Basis)	Control	Sorbitol	Maltitol
Wheat flour	63.4	63.4	63.4
Sucrose	16.3	-	-
Sorbitol	-	16.3	-
Maltitol	-	-	16.3
Butter	13.3	13.3	13.3
Water	6.1	6.1	6.1
Salt	0.5	0.5	0.5
Leavening agents	0.4	0.4	0.4

**Table 2:** Dough pieces and biscuit morphology attributes.

Formulation	Raw Diameter (cm)	Baked Diameter (cm)	Shrinkage Index (S)	Raw Thickness (cm)	Baked Thickness (cm)	Thickness Gain (cm)
Control	6.00 ± 0.02 ^a^	5.95 ± 0.03 ^a^	0.7 ± 0.8 ^b^	0.38 ± 0.03 ^a^	0.65 ± 0.02 ^a^	0.27 ± 0.03 ^a^
Sorbitol	5.98 ± 0.02 ^a^	5.91 ± 0.03 ^b^	1.2 ± 0.5 ^a^	0.40 ± 0.01 ^a^	0.59 ± 0.01 ^b^	0.20 ± 0.01 ^b^
Maltitol	5.93 ± 0.03 ^b^	5.88 ± 0.02 ^c^	1.0 ± 0.6 ^ab^	0.39 ± 0.01 ^a^	0.58 ± 0.01 ^b^	0.19 ± 0.01 ^b^

Results are expressed as mean and standard deviation (n = 15). Mean values with the same superscript letter within the same column are not significantly different at *p* < 0.05.

**Table 3:** Water content (% d.b.) of dough, biscuit centre and biscuit periphery.

Formulation	Dough Water Content (%)	Biscuit Centre Water Content (%)	Biscuit Periphery Water Content (%)
Control	20.9 ± 0.3 ^b^	2.5 ± 0.1 ^b^	0.75 ± 0.34 ^b^
Sorbitol	21.9 ± 0.2 ^a^	2.0 ± 0.6 ^b^	0.98 ± 0.31 ^b^
Maltitol	20.9 ± 0.4 ^b^	3.5 ± 0.5 ^a^	1.5 ± 0.4 ^a^

Results are expressed as mean and standard deviation (n = 3 for dough water content and n = 6 for biscuit water content.) Mean values with the same superscript letter within the same column are not significantly different at *p* < 0.05.

**Table 4:** Biscuit colour expressed as L*a*b* values and colorimetric distances between standard and substituted biscuits Δ*E*.

Location/Formulation	L*	+a*	+b*	Δ*E*
Upper Surface—Centre				
Control	72 ± 2 ^ab^	7 ± 1 ^b^	31.1 ± 0.7 ^a^	0
Sorbitol	71 ± 2 ^b^	8 ± 1 ^a^	29.7 ± 1 ^b^	2.4
Maltitol	74 ± 3 ^a^	7 ± 1 ^b^	28.6 ± 0.8 ^c^	2.7
Upper Surface—Edge				
Control	67 ± 3 ^a^	9 ± 1 ^ab^	30.8 ± 0.7 ^a^	0
Sorbitol	62 ± 3 ^b^	10 ± 1 ^a^	27 ± 1 ^c^	6.4
Maltitol	67 ± 2 ^a^	9.0 ± 0.4 ^b^	28 ± 1 ^b^	2.8
Lower Surface—Centre				
Control	78 ± 1 ^a^	0.7 ± 0.3 ^c^	24 ± 1 ^c^	0
Sorbitol	68 ± 3 ^c^	9 ± 1 ^a^	28.8 ± 0.9 ^a^	14
Maltitol	74 ± 4 ^b^	5 ± 3 ^b^	27 ± 1 ^b^	6.5
Lower Surface—Edge				
Control	63 ± 5 ^b^	11 ± 2 ^ab^	31 ± 1 ^a^	0
Sorbitol	61 ± 2 ^b^	11.2 ± 0.6 ^a^	28.2 ± 1.4 ^b^	3.5
Maltitol	66.0 ± 1.7 ^a^	9.7 ± 0.3 ^b^	29 ± 1 ^b^	3.8

Results are expressed as mean and standard deviation (n = 15). Mean values with the same superscript letter within the same biscuit location and column are not significantly different at *p* < 0.05.

**Table 5:** Biscuit hardness (N) measured at 1 h and 2 h after baking and porosity (%).

Formulation	Hardness 1 h after Baking (N)	Hardness 2 h after Baking (N)	Porosity Pt (%)
Control	25 ± 4 ^b^	25± 6 ^a^	55 ± 2 ^a^
Sorbitol	29 ± 4 ^a^	29 ± 3 ^a^	51 ± 2 ^b^
Maltitol	29 ± 4 ^a^	29 ± 5 ^a^	50 ± 1 ^b^

Results are expressed as mean and standard deviation (n = 9 for hardness and n = 15 for porosity). Mean values with the same superscript letter within the same column are not significantly different at *p* < 0.05.

**Table 6:** Mean hedonic scores of biscuits (n = 62). A 9-point hedonic scale was used for liking of biscuit (1 = dislike extremely, 5 = neither like nor dislike, 9 = like extremely).

Formulation	Mean Hedonic Score
Control	6.56 ± 1.38 ^a^
Sorbitol	4.55 ± 1.44 ^c^
Maltitol	5.50 ± 1.44 ^b^

Mean values with the same superscript letter are not significantly different at *p* < 0.05.

## Data Availability

Data are available on request from the corresponding author except for sensory analysis data unavailable due to privacy constraints.
